# Biopolymers for Biological Control of Plant Pathogens: Advances in Microencapsulation of Beneficial Microorganisms

**DOI:** 10.3390/polym13121938

**Published:** 2021-06-10

**Authors:** Roohallah Saberi-Riseh, Mojde Moradi-Pour, Reza Mohammadinejad, Vijay Kumar Thakur

**Affiliations:** 1 Department of Plant Protection, Faculty of Agriculture, Vali-e-Asr University of Rafsanjan, Rafsanjan 7718893514, Iran; moradi.mojde21@gmail.com; 2 Research Center of Tropical and Infectious Diseases, Kerman University of Medical Sciences, Kerman 7618866749, Iran; r.mohammadinejad@kmu.ac.ir; 3 Biorefining and Advanced Materials Research Center, Scotland’s Rural College (SRUC), Kings Buildings, West Mains Road, Edinburgh EH9 3JG, UK

**Keywords:** agriculture, biopolymer, encapsulation, formulation

## Abstract

The use of biofertilizers, including biocontrol agents such as Pseudomonas and Bacillus in agriculture can increase soil characteristics and plant acquisition of nutrients and enhancement the efficiency of manure and mineral fertilizer. Despite the problems that liquid and solid formulations have in maintaining the viability of microbial agents, encapsulation can improve their application with extended shelf-life, and controlled release from formulations. Research into novel formulation methods especially encapsulation techniques has increased in recent years due to the mounting demand for microbial biological control. The application of polymeric materials in agriculture has developed recently as a replacement for traditional materials and considered an improvement in technological processes in the growing of crops. This study aims to overview of types of biopolymers and methods used for encapsulation of living biological control agents, especially microbial organisms.

## 1. Introduction

Despite the background of unsustainable population growth and dramatic changes to the ’world’s climate, we somehow have to find a way to feed people around the world, both now and in the future, without further destroying our planet. Given that agriculture is the industry that supplies major raw ingredients of the food industry, finding ways to increase production and improving the quality of agriculture products are important. Today, environmental destructive effects due to overuse and incorrect use of chemical pesticides, have led to an increase in biological control used in most parts of the world [1]. The application of plant growth-promoting rhizobacteria (PGPR) has been used for sustainable agriculture production and recovery of degraded lands [2]. Since biological control has a key role in controlling diseases, it has received more attention in recent years.

**Figure 1:**
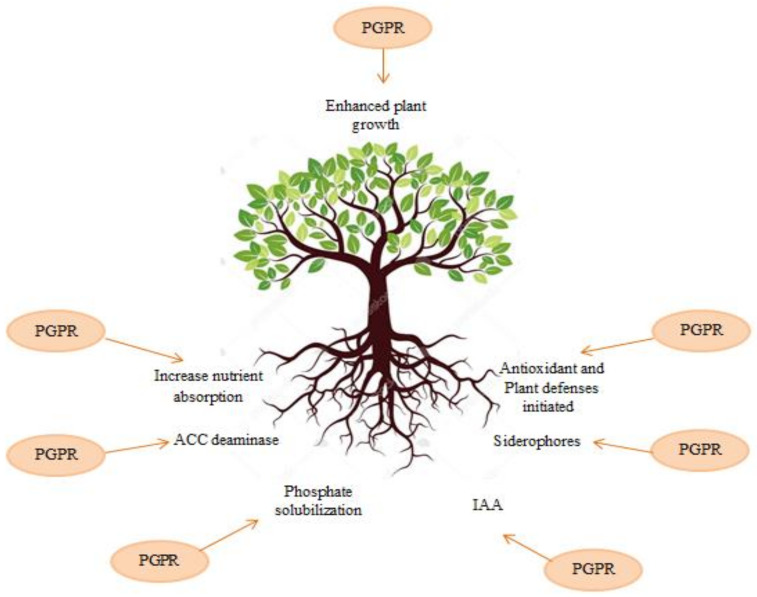
Mechanisms of plant growth-promoting rhizobacteria (PGPR) based growth and health improvement in plant Reproduced from [10].

Over the years, the application of microbial toxins in agriculture has increased [3]. Bio-control can play a main role in the development of crop production [4]. However, a high percentage of studies on beneficial soil bacteria have focused on bacterial physiology characteristics and genetics, and research on microbial formulations accounts for less than one percent of scientific study on microorganisms [5]. Beneficial microorganisms isolated from agricultural lands and crops can lead to the decomposition of organic matter residues, suppress plant diseases and pathogens in the soil, and strengthen the cycle of biologically active compounds such as hormones, enzymes, and vitamins (Figure 1) [6,7,8,9].

The biological control of soil-borne plant pathogens, by antagonistic bacteria is often unstable [11]. One of the main reasons for the instability of the beneficial effects of antagonistic bacteria is their inability to effectively and sufficiently colonize the roots [12]. This can be related to the characteristics of the bacterium or environmental factors, both biotic and abiotic [13]. The use of free bacteria for colonization of plant roots is not natural, because microbial agents are sensitive to several changes such as temperature, pH fluctuations, humidity, and environmental stresses [14]. Alginate, chitosan and starch are biodegradability and biocompatibility polysaccharides that are safe for human, and widely used for different sciences especially agriculture [15].

Despite the increasing information on how biological control agents work, their practical use on the farm to control the disease is often found to be difficult. One of the reasons for this failure is that biological control products are consumed in the same way as chemical products [16]. The effectiveness of the biocontrol agent requires its use at the right time and place because secondary metabolites are not produced in large amounts and are unable to move long-distance. Therefore, biocontrol agents must be in contact with the pathogen at a specific location [17].

**Figure 2:**
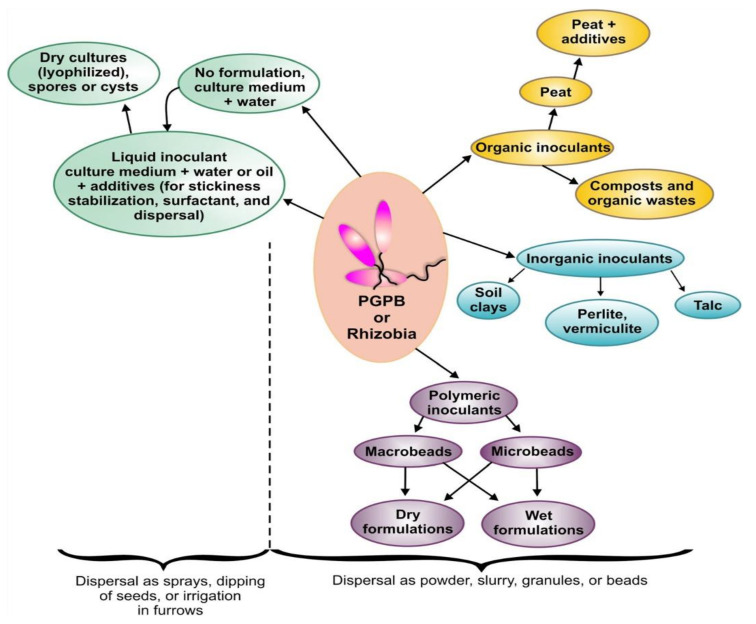
Formulations of plant rhizobacteria for using in agriculture. Reprinted with permission from Springer, Plant and Soil [18], Copyright 2014.

Inoculant carriers can fall into five categories: (1) Plain lyophilized microbial cultures (2) Liquid inoculants, with some Chemical additives to better stickiness, stabilization, function, and dispersal (3) Inert materials: polymers (4) Waste products of industrial and agriculture resources, such as Lignin, (5) Soils: coal, clays, peat and inorganic soil (Figure 2) [18].

**Figure 3:**
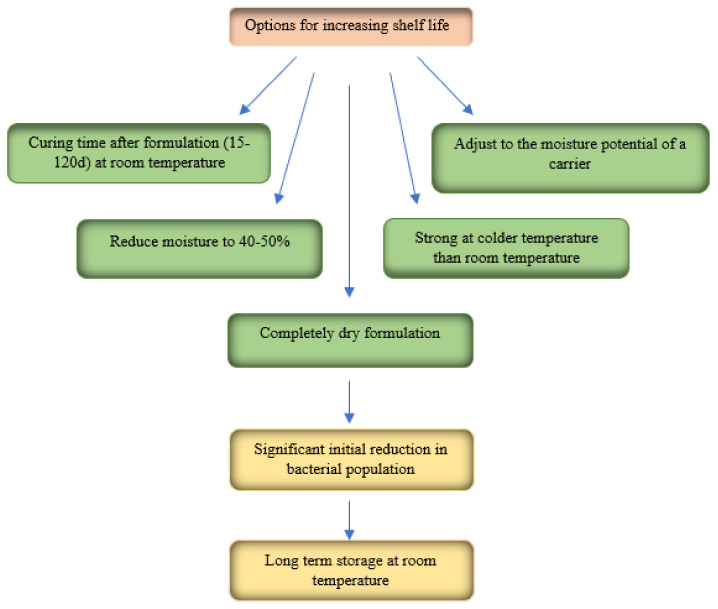
Improving shelf life of inoculants. Reproduced from [18].

To use inoculants produced of organic and inorganic materials in agriculture, a storage period between the production of the formulation and the time of its use is required. Improving the shelf life of the inoculant while holding its biological traits intact, is an important aspect in formulation technique (Figure 3). One of the most important suggestions to this essential problem have been directed to decrease formulation moisture or storing at cooler temperatures [5].

Conventional biopesticides and biofertilizer are used in plant applications as liquid (including cell suspensions in water, oils, and emulsions), powder (such as wettable powders, dust, and granules) [19]. The results of Lee et al. [20] showed the wettable powder formulation *Bacillus licheniformis* N1, based on corn starch and olive oil, can successfully control Botrytis grey mold in the greenhouse condition. Numerous studies have indicated the potential of bacterial strains for controlling multiple diseases happening on a broad range of plant species [21]. Bacillus species, especially *Bacillus subtilis* and *fluorescent Pseudomonas* have been used to control a variety of plant pathogens, such as *Alternaria dauci* on Carrot [22], *Macrophomina phaseolina* on Chir-pine [23], *F. oxysporum* on Cotton [24], *R. solanacearum* on Tobacco [25] and *Phytophthora drechsleri* on pistachio [14]. In recent years, the methods of optimal use of biocontrol agents have been increasingly considered under the title of controlled release [26]. The survival of the biocontrol agent and its effectiveness in controlling pests and pathogens largely depends on the material used to formulation them. The use of polymers such as alginate, starch, chitosan, etc., in the formulation of biocontrol agents, has been investigated [19,27].

With the help of some regulators, the release of biocontrol agents can be adjusted. The release could be delayed or accelerated [28]. Controlled release systems come in many forms. New science has produced many active substances for human life and health, the use of which is usually ineffective due to the inability to transfer them to the target at the right time and in the right amount. This method provides the active chemical ingredients available to achieve the goal at the required speed and time [29]. The basic formulations of controlled release systems include active materials and a carrier [30]. This is where the issue of polymeric materials in controlled release systems, comes into play. It is important to discuss the formulation and commercialization of biocontrol products to implement laboratory results. One of the most important factors in the success of a biological control agent is its ability to rapidly growing the population, which must be considered in its formulation. The formulation of products is changing day today, with of providing conditions for more stable antagonist factors in nature, so that moisture and nutrients are not removed from the access of bio-control agents. Also, the storage period and transportation conditions, in formulation and packaging should be considered. A suitable formulation for biological control agents is more difficult and expensive than chemical pesticides. Therefore, to develop the application of biological control method, it is necessary to develop the importance of environmental health.

## 2. Mechanisms of PGPR Bacteria on Biocontrol of Plant Pathogens

PGPR can interact with the plant and increase plant growth as a result of the metabolites that they release in the rhizosphere [31,32]. PGPR can stimulate plant growth via a wide range of mechanisms such as the synthesis of materials that can be assimilated directly by plants [33], the production of nutrients, induce resistance and, the prevention of plant diseases [34]. Biocontrol bacteria use various mechanisms like fixation of nitrogen, solubilization of mineral phosphate production of auxin, siderophore, ACC deaminase, hydrogen cyanide, production of various enzymes (lipase, protease, cellulase, chitinase, etc.) to improve plant growth and plant disease management [34,35,36,37].

Rhizobia bacteria able to produce siderophores have been reported as potential biofertilizers, increasing the production of lettuce, carrot, tomato, pepper, strawberry and chickpea. Ghavami et al. [38] reported several bacteria by produce siderophores, that contributed to improved canola and maize plant growth.

Phosphorous is the necessary nutrient for plants. Phosphorous is quite insoluble in soils and as a result, chemical phosphorous fertilizers were practical in traditional agriculture for the deficiency of this element [39]. By the way, when used practically as chemical fertilizers to crop fields, phosphorous passes quickly to become insoluble and unavailable to plants. So, the use of phosphorous solubilizing bacteria might represent a green replacement for these environments damaging chemical phosphorous fertilizers. According to the results of Liddycoat et al. [40] indicated that pseudomonas strains can increased asparagus seed germination and growth factor under conditions generated in greenhouse conditions. The mechanisms of PGPR to plant disease prevention Includes the production of antibiotics, cell wall degrading enzymes and siderophores [41]. Moradi-Pour et al. [14] indicated the ability of *B. subtilis* bacteria to controlled of pistachio gummosis and improve plant growth. also, reported *P. fluorescens* strain reduced the infection of *F. solani* on potato [42].

## 3. Encapsulation

**Figure 4:**
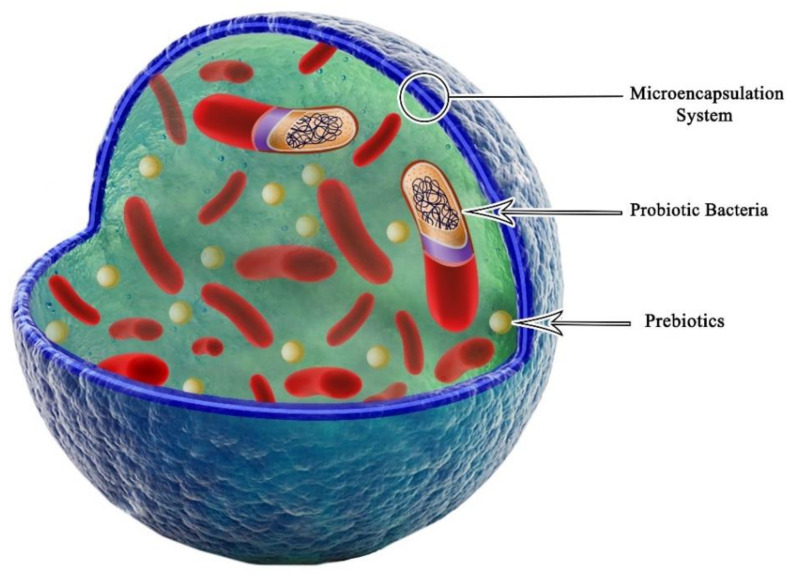
Schematic representation of bacterial encapsulation Reproduced from [50].

Encapsulation is a physical or chemical process to produce beads with size ranges from a few nm to a few mm (Figure 4) [43,44]. Encapsulation techniques of bacteria causes the production of a physical barrier between the internal material and its around to protect them against environmental condition such as moisture variations, pH alterations, and oxidation [45]. This method has advantages, including protection of encapsulated materials against environmental changes as well as the controlled release of materials [46]. The most important purpose of biological agents’ encapsulation is to improve its stability in the process of use [47]. Capsule materials are polymers that used for capsule matrix and often, the capsule will be including 1–5 additives with synergistic functional to further improve crops properties [27].

The survival of encapsulated cells depends on the type and concentration of coatings, capsule size, the initial number of cells, and the type of bacteria. The capsule wall can contain one or more substances. The capsule wall is designed to prevent the release of nuclear material to the outside environment until the perfect time. This method is used to protect sensitive food elements such as flavors, vitamins, or salt, from water, oxygen, or light. It also liquids that are difficult to work and transform into a powder that is easily immersed in water. it protects certain elements from other nutrients in food during storage. Encapsulation of micro-organisms is described as the packaging method of solids, liquids, or gaseous material on a smaller scale. The contents of capsules release in special conditions [42]. Microcapsules are composed of a semi-permeable, spherical, thin with strong membrane around a nucleus that can be solid or liquid [48]. The encapsulation of microorganisms in polymers is a branch of bacterial carrier technology. Recently, as a new approach, intelligent polymers have been used to encapsulate some chemicals compounds, such as antibiotics, and about their controlled release into the body, very promising research has been done [49].

Solid and liquid formulations of biological products and application of traditional methods face limitations such as short shelf life, lack of suitable carriers, transportation, and storage problems [51]. Therefore, achieving a new formulation and commercializing bio-fertilizers can be very useful [52]. The use of liquid formulations causes direct contact of the inoculation agent with the plant roots, and consequently, the survival of bacteria in the roots increases. However, the survival rate of bacteria decreases rapidly [18]. The use of liquid formulations for long-term storage and survival of cells requires special conditions. A formulation contains one or more microorganisms, such as beneficial bacterial species that are easy to use and affordable. Several experiments have been performed on different polymers for use in the formulation of bacteria. Encapsulation of microorganisms can protect them from biotic and abiotic stresses [53]. It is one of the novels and most efficient techniques. The encapsulated cells, with the controlled system, can slowly release the microorganisms into the soil and have longer effectiveness [51]. In fact, encapsulation tends to stabilize and maintain cells against biotic and abiotic soil stresses. Several studies have pointed to various encapsulation techniques to increase the viability of microbial (Table 1), [18,30,52,53,54,55].

**Figure 5:**
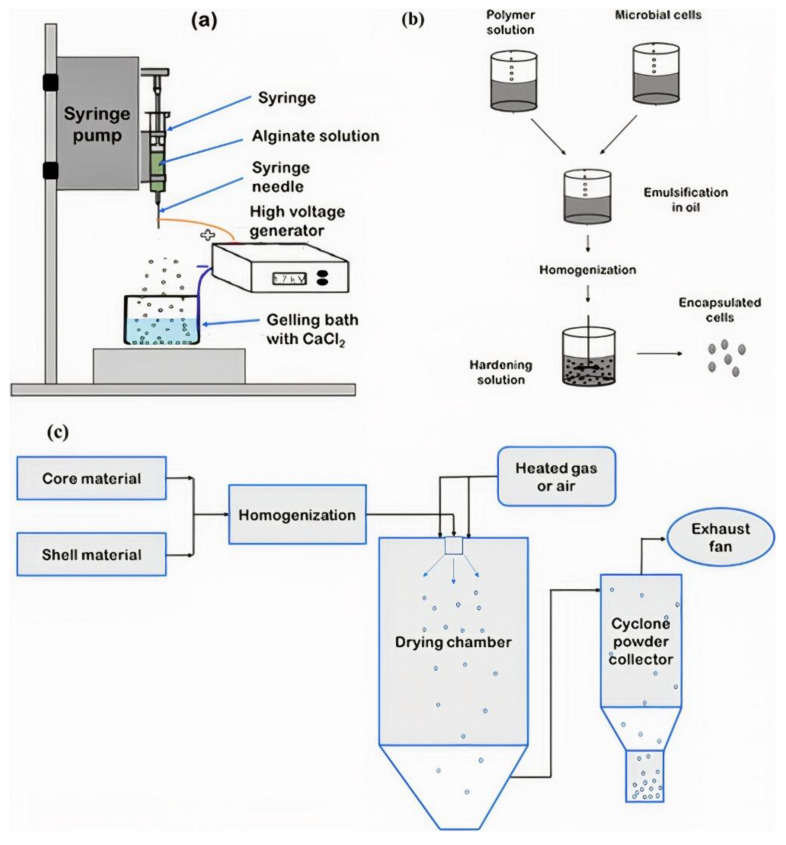
Schematic representation of encapsulation methods: (**a**) Extrusion (**b**) Emulsion (**c**) Spray drying. Reprinted with permission from Elsevier, Food Bioscience [81], Copyright 2018.

Encapsulation of material into carriers can be achieved by various technology such as emulsification, lyophilization, extrusion coating, spray chilling, fluidized-bed coating, coacervation, spray drying, and thermal gelation. Various factors, including the physio-chemical properties of coating and core materials and its application, are effective in choosing the right encapsulation process. Among them, spray drying, emulsion and extrusion are effectively applied for microencapsulation of plant growth-promoting bacteria (Figure 5) [70].

### 3.1. Spray Drying

This technique is the most common method widely used to encapsulate of probiotic cells in the food industry. In this industry, this method is applied for the protection of microbes [71,72]. In this method, living cells are dissolved in polymeric matrices. The polymeric matrix can be gum Arabic and starch because they tend to form spherical particles when dried [73]. The advantage of this method is the high speed, rapidity and reproducibility, ease of operation, large-scale implementation and low price of products [74,75]. The main disadvantage of this method is the use of high temperatures causing a reduction in bacterial survival rate [76] and difficult to use for small applications [77]. Boza et al. [56] used the spray drying technique for encapsulation of *Beijerinckia* sp. cells. Spray drying methods are suitable for using of PGPR [59,60,78]. The ability of survival rate in this process depends on the bacterial strain. For example, spray-dried *Lactobacillus acidophilus* and *Bifidobacterium breve* indicated a survival rate of only 76% and 26%, respectively [57,58].

### 3.2. Emulsion

The emulsion is a chemical method for the encapsulation of living cells. An emulsifier, a surfactant, and a hardening agent (such as CaCl_2_) are required for encapsulation by emulsification [55]. This method can be used to keep bacteria alive on a larger scale [79]. The bead size in this method is determined by stirring as well as changing the water and oil ratio [80]. The emulsion process gives a high survival rate of the bacterial strain and is simple to scale up. Adding the gel beads into the second polymer solution increases the encapsulation efficiency in two ways: 1: Creating a coating layer for extra protection 2: probably give amended organoleptic properties [80]. This method gives spherical and water-insoluble particles [70]. Emulsification is applied to produce a formulation with suitable shelf-life in biological control. Moradi-Pour et al. [42] stated that the encapsulation of *Pseudomonas fluorescent* T17-4 and VUPF5 using alginates and gelatin has excellent stability at room temperature up to 6 months of storage and improved survival under greenhouse conditions. Holkem et al. [62] produced microcapsules containing *Bifidobacterium* BB-12 by emulsification.

### 3.3. Extrusion

Extrusion is a physical method for the encapsulation of living cells [81]. In this method, the solution containing the living cell enters calcium chloride solution through a high-pressure nozzle [79]. Extrusion is a cheap and simple technique in which no damage to the bacterial cell is observed and has a long shelf life [82]. The bead size depends on the distance between the hardening solution and syringe, viscosity, concentration polymer type, and diameter of the syringe needles [83]. This technique does not contain detrimental solvents and can be used in anaerobic and aerobic conditions [70]. *B. subtilis* VRU1 encapsulated in alginate-bentonite with extrusion showed an improved survival rate under greenhouse conditions [53]. In this method, no harmful solvents are used, and it can also be used in different conditions [84]. The problems of the extrusion technique are the difficulty of using it on a large scale and the slow formation of beads. *B. cereus* C1L encapsulated in maltodextrin and gum arabic by extrusion method showed high efficiency to control leaf blight in corn [63].

## 4. Polymeric Materials for Cell Microencapsulation

**Figure 6:**
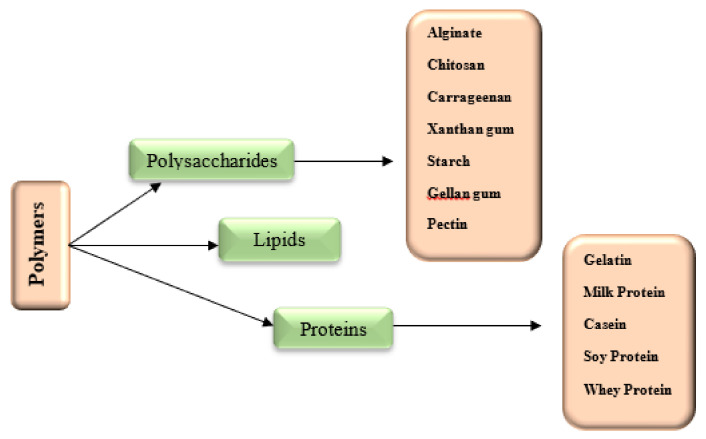
Classification of biopolymers usable in encapsulation processes. Reproduced from [15].

Various carriers (including carbohydrates and proteins) are used for the encapsulation process, typically obtained from algae (κ-carrageenan and alginate) and other plants (gum Arabic and starch) or animal proteins (gelatin) (Figure 6).

### 4.1. Alginate

**Figure 7:**
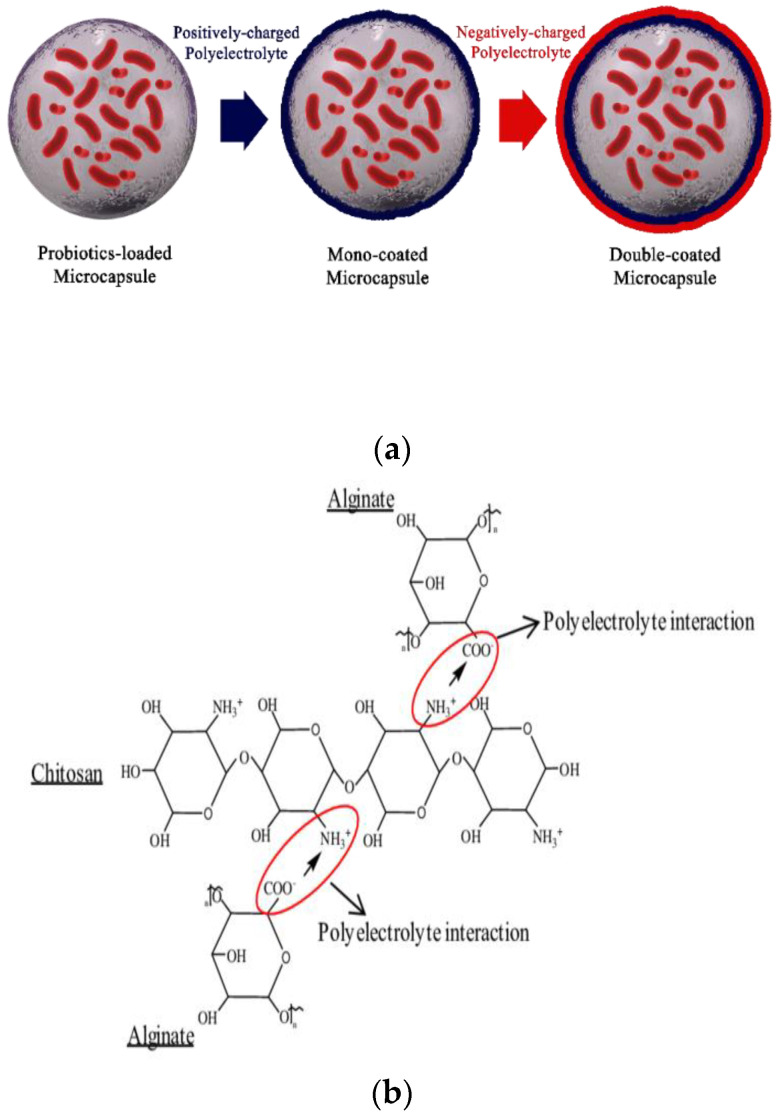
(**a**) The layer-by-layer method design on bacterial microcapsules via coatings [50], (**b**) Ionic interaction between chitosan and alginate [83].

Alginate is one of the most important biopolymers [85], it becomes a multifunctional component in different science, it included in a group of compositions that are commonly considered safe. Alginate is linear heteropolysaccharides, the property of which, as a preservative, depends on the combination of β-D-mannuronic acid and α-L-guluronic acid, which leads to the binding of subunits and gel formation. Commercial alginates are obtained from various species of brown algae [86]. Also, this material can be synthesized by *Pseudomonas* species and *Azotobacter vinelandii* [87]. Alginates are the esters or salts of alginic acids. Entrapment and Immobilization of PGPR bacteria in these polysaccharides is possible due to its non-toxic, quick method for bacterial cells. The viscosity of the sodium alginate will enhancement with the number of monomeric units (length of the macromolecule). This material is used in different fields including agriculture, medicine and food. The biggest advantage of alginates is the gel matrix is easily formed around the cells, it is cheap, biocompatibility, cost-efficiency, non-toxicity [79] and safe to the body. The disadvantages of alginate beads are that they are not compatible with alkaline conditions [88]. However, the faults of this polymer can be atoned by mixing alginate with various biopolymer, and coating the capsules with another polymer such as gelatin [89] and chitosan, for example in (Figure 7a) was demonstrated more details about this process, where primary microcapsules produced by alginate was consecutively coated by chitosan [50]. Therefore, as stated that strong ionic interactions between the anionic group (alginate) and cationic group (chitosan) (showed in Figure 7b), cause improved effective protection and capsule stability. Simple control of released microorganisms in the soil and high durability at room temperature has led to the acceptance of alginate granules in agriculture to combat plant pathogens [90]. Many studies are reporting that mixture of chitosan-alginate for encapsulation of PGPR bacteria allowed better viability, among them we can mention [91,92]. According to the research of Zou et al. [64], the encapsulation of *Bifidobacterium bifidum* F-35 in alginate was reinforced with the addition of starch/pectin or coating with chitosan/PLL to increase its survival for bacteria.

### 4.2. Chitosan

**Figure 8:**
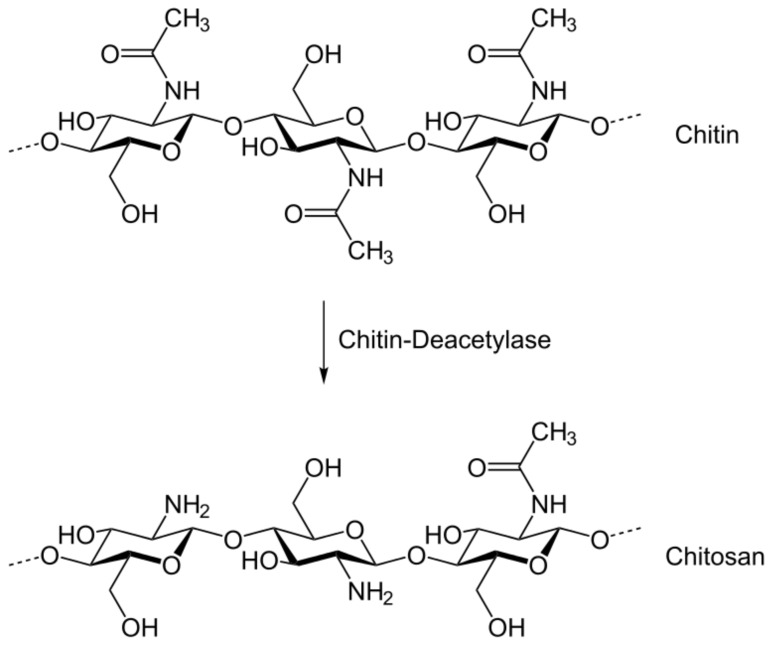
Comparison among the chemical structures of fully deacetylate chitosan and fully acetylated chitin Reproduced from [105].

Chitosan is a second natural polysaccharide [93], It is the deacetylated derivative of chitin and chemically composed of replicates of D-glucosamine and N-acetyl-D-glucosamine units (Figure 8) [94]. Chitin is the structural component of the external skeleton of arthropods such as shrimp and crab shell, the fungal cell walls and insects [93,94,95].

Chitosan is a non-toxic, biodegradable and biocompatible biopolymer [96]. Chitosan has versatile mechanical properties, which have led to its enhancement use in different applications such as encapsulation technology, controlled release coatings, drug delivery system, nanofiltration, and tissue engineering [15,97,98]. Chitosan is soluble in neutral and acidic media, but the viscosity and solvability of the solution are dependent on the degree of acetylation and length of chains. This material has a positively charged and it forms ionic hydrogels by the addition of negatively charged polymers such as alginate [99] and xanthan [100] and is used in various sciences, such as agriculture, food and medicine due to biodegradability and biocompatibility [15]. Ability to increase chitosan permeation for the first time described by Illum et al. in 1994 [101]. The main mechanism of this permeability increases to be based on the positive charges of the chitosan [102]. Characterization of chitosan makes it ideal for use as a material for encapsulation [103]. Encapsulation of PGPR bacteria with alginate and its coating by chitosan might be a beneficial technique to enhancement the survival of bacteria in acidic conditions [104]. In a study by Li et al. [65] *Lactobacillus casei* ATCC 393 was encapsulated in matrices of alginate, chitosan, and carboxymethyl chitosan with extrusion technique, and the result showed enhancement the cells’ viability up to 10^8^ cfu/g after 28 days of storage at 4 ˚C.

### 4.3. Starch

**Figure 9:**
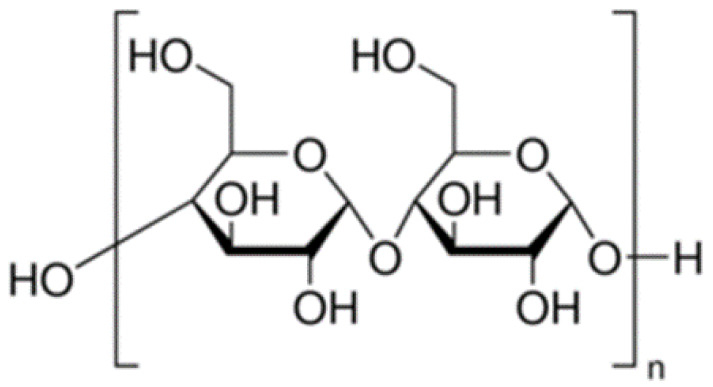
Chemical structure of starch [112].

This polymer is one of the main abundant biopolymers produced by all plants and has many benefits, its cheap and non-allergenic. Today, used starch in modified and native forms to encapsulate of PGPR bacteria [106]. Its chemical structure has compound α-D-glucose units linked by glycosidic bonds (Figure 9). Starch has extraordinary properties such as biocompatibility, biodegradability, its cheap, edible, abundant and cost-effectiveness, therefore has a long story in PGPR formulation [107]. Starch is insoluble and dense, and its hydrate formation only in at natural temperature in water. The structure of this polymer is irreversibly lost when this polymer is warmed about 80 ºC in water. Enzymes such as amylases use to hydrolyses the starch granules [99]. chemically modified and hydrolysates starch are used as encapsulation matrices [108]. Various materials such as insecticides, fungicides, herbicides, nematicides, bacteria, drug, etc., may be encapsulated in the starch matrix [109]. Starch has been used for encapsulation of and the results indicated high maintenance of ascorbic acid during storage [110]. Some PGPR bacteria can able to stick to this polymer but a few of researches for encapsulating PGPR by starch were reported. Among the polymers used in research of Lian et al. [111] to encapsulate tested, starch and gelatin were the suitable roles in protection of beneficial microbes in acidic condition. The mixture of starch, bentonite and alginate was a very effective mix for encapsulation and protection *Raoultella planticola* Rs-2 under biotic and abiotic conditions [68]. The encapsulation of *Bacillus subtilis* Vru1 in starch combination with bentonite and alginate with extrusion method, the results indicated the survival of this strain in capsules compared the bacterial without coating, was higher. Encapsulated bacteria have a better characterization and controlled *Rhizoctonia solani* on the bean, under in-vitro and in-vivo condition [53].

### 4.4. Pectin

**Figure 10:**
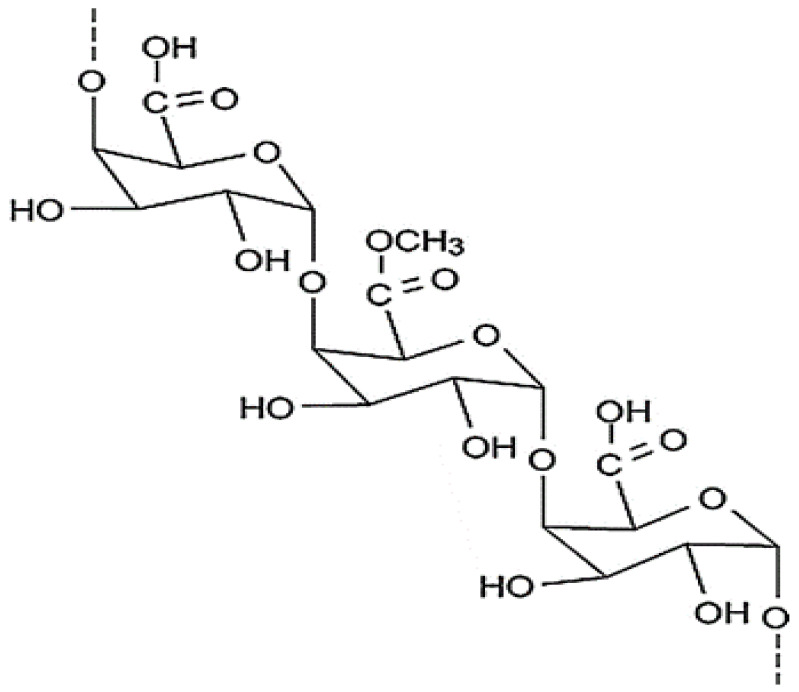
Chemical structure of pectin [119].

Pectin is water-soluble polysaccharides obtained from the cell wall of many products such as vegetable and fruit (Figure 10). It’s an anionic biopolymer, and an important polymer used in the encapsulation. Pectin is usually formed of pectinic acids, which are gels that could be formed when exposed to the correct conditions [113]. This material is a high molecular weight macromolecule, which can be converted into a hydrogel, have a flexible network of polymeric chains that can swell without dissolving in water [114]. The result of Bekhit et al. [115] encapsulated *Lactococcus lactis* subsp. Lactis in alginate/pectin microcapsules. In the study by Sun et al. [116] was indicated that *Lactobacillus delbrueckii* strains encapsulated in soy protein/pectin beads can increase bacterial viability compared with the control. Biopolymer mixtures are an excellent strategy for controlled release to enhancement formulation stability, the amount of core material in the matrix and to target specific. Pectin and alginate mixture indicated interesting synergistic characteristics than biopolymers alone [117]. Islan et al., reported Ciprofloxacin encapsulation in the alginate-pectin beads with controlled release in a gastrointestinal tract could be avoiding the Ciprofloxacin officious side effects during absorption [118].

### 4.5. Gelatin

**Figure 11:**
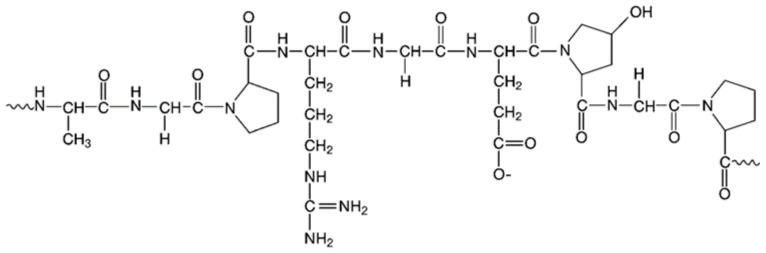
Chemical structure of gelatin [126].

Gelatin is a protein obtained from denatured collagen for example in skin, tendons, and bones that including high levels of glycine, hydroxyproline and proline (Figure 11) and can be used alone or in combination with other substances for PGPR encapsulation. The application of gelatin is limited because of its low network stability. However, its physical characteristics can be amended through the addition of cross-linking agents [120]. This material has an amphoteric nature; therefore, it is a suitable polymer for mixture with anionic carbohydrates like carboxymethyl cellulose, alginate, etc. [121] Due to the neutrality of gelatin, it is the most suitable option for reaction with gellan gum [122]. Gelatin is the most widely used biodegradable hydrogel. Mixtures of polysaccharides and proteins in PGPR bacteria encapsulation are practical due to their biocompatibility [123]. Gelatin is a non-toxicity material with biocompatibility and has a good membrane-forming ability [124,125].

### 4.6. Milk Proteins

**Figure 12:**
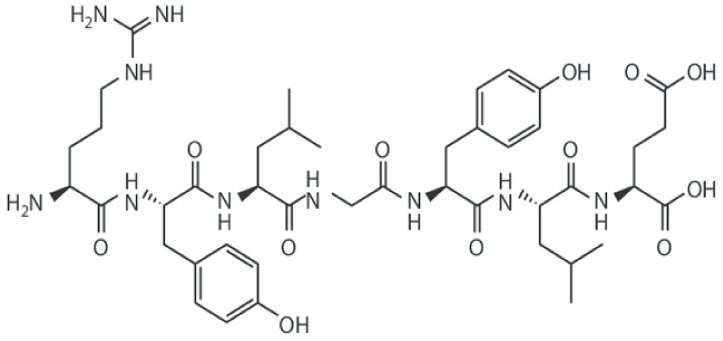
Chemical structure of milk proteins [127].

In the encapsulation technique, milk proteins are sometimes used [76]. Milk proteins, due to their physical and chemical properties (Figure 12), are good effective materials for the encapsulation of PGPR. The excellent jelly-like properties and good biological adaptations have made them a good option for encapsulating PGPR [84]. Some authors showed PGPR bacteria such as *L. rhamnosus* GG, B. L. *paracasei* F19 encapsulated in milk proteins [76].

### 4.7. Xanthan and Gellan Gum

**Figure 13:**
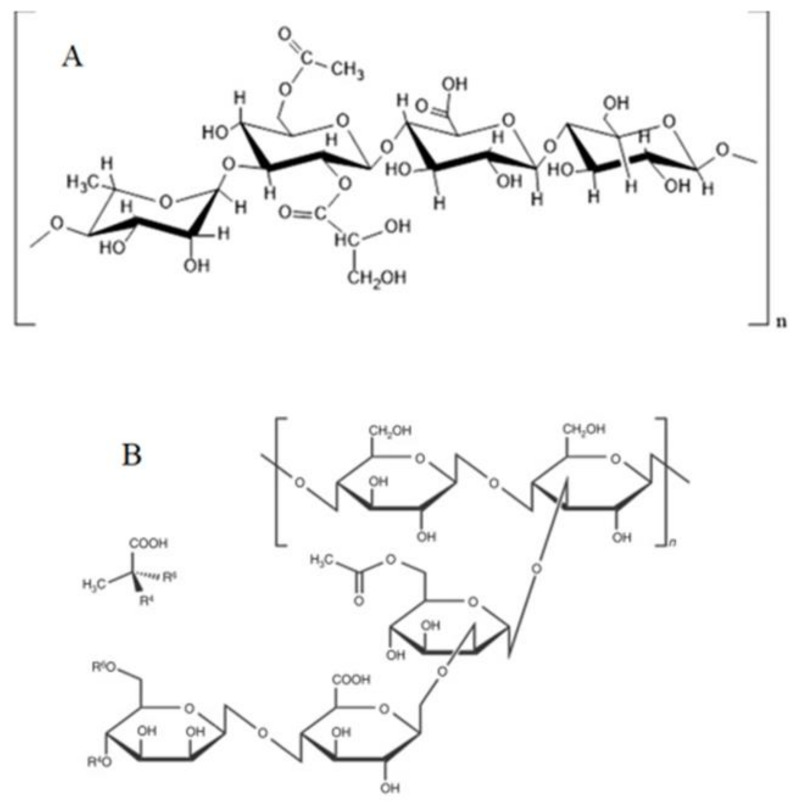
Molecular structure of (**A**) Gellan gum [129] and (**B**) Xanthan gum Reproduced from [130].

Gellan gum is a polysaccharide produced by *Pseudomonas elodea* and composed of one glucoronic acid, one rhamnose and two units of gucose (Figure 13A) [43,44]. The gellan gums gels with low acyl quantity requirement the presence of divalent stabilizing cations. One of its disadvantages is having a high gel setting temperature (about 1 h at 80–90 °C), which leads to the injuries of bacteria [121]. Xanthan is a heteropolysaccharide produced by *Xanthomonas campestris*, including repeated pentasaccharide units organized by one glucoronic acid unit, two mannose units and two glucose units (Figure 13B). This gum is dissolved in cold water and hydrates quickly. A mixture of xanthan–gellan gum can be used to encapsulate PGPR [43,44,128], the granules produced from a mixture xanthan–gellan gum are resistant to acidic conditions, unlike the granules obtained from alginates [70]. Saberi-Riseh and Moradi-Pour [61] encapsulated *Streptomyces fulvissimus* Uts22 in chitosan-gellan gum beads.

### 4.8. κ-Carrageenan

**Figure 14:**
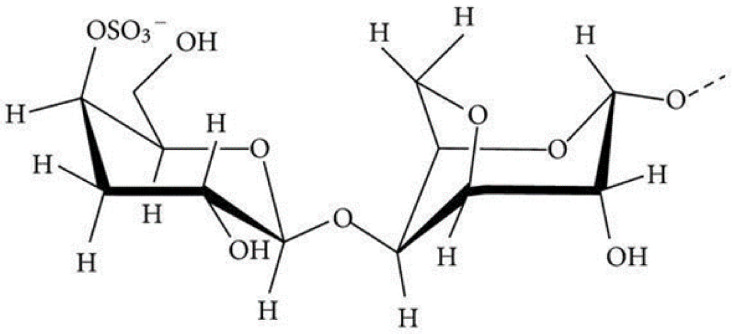
Chemical structure of κ-carrageenan [134].

κ-carrageenan is a series of sulfated linear polysaccharides (Figure 14) extracted from red seaweed [131]. Due to its excellent physical and chemical properties, it is commonly used in cosmetic, pharmaceutical and food industries, as a thickener and stabilizer as well as a jelly-causing agent [132]. This substance is in the form of powder, and it must be in the form of a solution to be used in the process of encapsulating materials, which requires heat it in the temperature range from 40–50 °C. Disadvantages of using κ-carrageenan for encapsulation of bacteria, is the relatively high temperature of the dissolution process that may lead to the death of the bacteria and the created gels are fragile [43,44]. Paᶊcalãu et al. [133] used calcium alginate - κ-carrageenan for acetylcholinesterase encapsulation.

### 4.9. Gum Arabic

**Figure 15:**
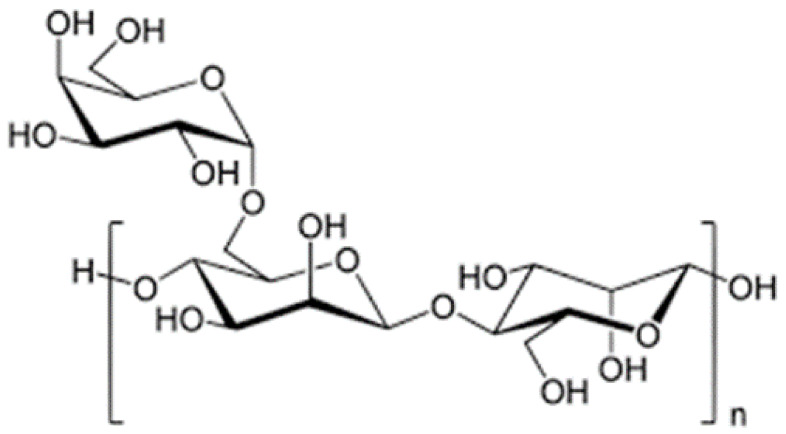
Chemical structure of Gum Arabic [141].

Recently, have been reported new biopolymers such as gum exudates in the encapsulation process [135,136]. Gum arabic is an exudation of plant from Acacia seyal and Acacia Senegal trees. Arabic gum due to its biocompatibility for in vitro and in vivo application, used as the carrier for bioactive molecules in encapsulation [137]. This gum a mixture of three various polymer fractions: carbohydrate-(arabinogalactan) (88%), high-protein (glycoprotein) (2%) and the low-protein (arabinogalactan protein complex) (10%) (Figure 15) [138]. These biopolymers are considered to be polymers with excellent possible for future applications of cell encapsulation [139]. The blend of gum arabic and maltodextrin have been widely used as wall materials in food ingredients encapsulation by spray drying technique. According to the result of Frascareli et al. [140] observed Arabic gum was effective in encapsulation of coffee oil. According to the study of Desmond et al. [66] reported the survival rate of spray-dried powders containing Arabic gum of *Lactobacillus paracasei* NFBC 338 was increased and they indicated that Arabic gum can protect bacteria during drying, storage [66].

### 4.10. Poly-L-Lysine

Poly-L-lysine is a poly amino acid, has a peptide bond along the α-carboxyl and ε-amino groups. It is produced by *Streptomyces albulus* [142] and been approved a food safe holder. Poly-L-lysine have a wide antimicrobial effect on bacteria, fungi [143] and used for encapsulation of food, fish, rice, cooked vegetables [144].

### 4.11. Poly l-Glutamic Acid

This is a natural polypeptide, non-toxic, hydrophilic and contain l-glutamic acid which linked by amide bonds, and it is produced by microbial species of Bacillus [145]. The high biodegradability Poly l-glutamic acid [146,147], make it as promising composite in encapsulation [148] and tissue engineering [149].

## 5. PGPR Encapsulation in Agriculture

In recent years, the number of PGPR encapsulation has increased in agricultural sectors. A team of researchers have using alginate and pea protein to encapsulate *Bacillus subtilis* B 26 [150]. *B. subtilis* was encapsulated using xanthan and carboxymethyl cellulose biopolymers indicated the significant decrease in *Meloidogyne incognita* compared to control under greenhouse conditions [151]. According to the results of He et al. [152] observed, *Pseudomonas putida* Rs-198 encapsulated in alginate-bentonite-starch have higher survival rate with effective colonization on the root of the cotton plant. The result of Slusarenko et al. [153] showed alginate encapsulation of garlic juice used in the soil-pot test, which decreased the *Phytophthora* infection in tomato seedlings. The survival of the *Bacillus megaterium* encapsulated in calcium alginate microcapsules was greater than the free cells when exposed to ultraviolet light and high-temperature treatment. Spraying rice plants with encapsulated *B. megaterium* under greenhouse conditions, indicated that the capsule form was more effective than chemical fungicide for suppressing rice sheath blight disease [154].

Microencapsulation of *Pantoea agglomerans* strain E325 and its potential for controlling *Erwinia amylovora* on apple and pear have been identified [155]. According to the result of Ma and Feng, [156] microencapsulation of *Bacillus subtilis* strain B99-2 had high potential of biological control of *Rhizoctonia solani* in roots of tomatoes. Gluten and gum arabic microcapsule of *Bacillus cereus* ANTI-8098A used for biocontrol of bacterial wilt disease and it was observed that this formulation reduced the risk of this disease by 93% [157].

In the biocontrol of plant pathogens with PGPR bacterial agents, achieving an efficient formulation is very important that if it is not suitable, the bacteria will quickly be out of reach plant and will be reduced the effective population. For this reason, one of the ideas that can be raised in this field is the issue of encapsulation, in which the bacteria, by control of release from the polymer wall of the capsule, increases its survival against environmental factors and causes better colonization of the plant. Since pistachio is the main important export yields of Iran, so to maintain its global position, new methods should be used in the management of pests and diseases. In a study, Moradi-Pour et al. showed that *Bacillus velezensis* alginate-gelatin nanocomposites were able to control up to 96.3% of pistachio gummosis and also increased pistachio growth (unpublished). As in the medical fields and drug delivery, it is observed that the typical release of drug in the body cause fluctuations in the amount of drug in the blood, sometimes between the toxic and therapeutic dose of medication. The therapeutic range of a drug should meet the patients needs until the next dose is given. However, it is observed that an increase or decrease in a drugs dose in the body affects its effectiveness. Agricultural chemicals such as herbicide, if used directly, will produce similar results. Also, significant efforts have been made to use the potential of nanotechnology in drug delivery at a particular location with small or large molecules and other active ingredients [158]. This technology has also been used in agriculture, for example, the use of controlled-release fertilizers can be more effective and useful than the usual methods of using fertilizers and nutrients, it also reduces nutrient losses, saves work, reduces stress or poisoning due to increasing the availability of nutrients and induces synergistic effects between specific chemical forms of nutrients on plants, and it reducing environmental pollution caused by excessive consumption of substances such as nitrate and phosphate [159,160].

Research on micro-and nanocapsules began around 1950 and expanded rapidly in the 1970s. In recent years, extensive studies have been conducted on the encapsulation of materials in the biological, chemical, biotechnological, pharmaceutical, and engineering sciences. Microencapsulated materials are performed mainly to prevent the chemical reaction between the active substance and the environment (ultraviolet light, oxygen, and moisture), to prevent the side effects of the active substance, prolonging the shelf life of the active substance, ease of use by solidification of the liquid core and controlled-release material [160]. This feature can also be used in agricultural operations and paint industries. To encapsulate the core material, various compounds can be used as a capsule shell. The most important factors in choosing material for capsule shells are the type of core material and its application after encapsulation [46]. Nanoparticles can be used to make fertilizers with the delayed controlled release that are more effective than conventional fertilizers. Researchers have tried to reduce the negative effects of sunlight by encapsulation of bacterial cells, and by producing encapsulated formulations of bacteria containing accompanying substances. They have been able to protect their spores and crystals from the effects of sunlight to some extent [68,156,157,158,159,160,161,162,163]. In recent years, some researchers have used biopolymers to encapsulate PGPR bacteria (Table 2).

## 6. Conclusions

Encapsulation of bacteria with various materials has been done in recent years and the progress that we have performed involves the recognition of different polymers that have excellent possible for encapsulation technology. Most biopolymers usage in bacterial encapsulation for agriculture is not characterized and reviewed. Therefore, we have studied biopolymers in the recent years and their usages in industries and their restrictions for the better understanding of the readers. And indicated the function of encapsulation of bacterial agents in the biocontrol of plant diseases. In the near future, encapsulation will purpose different active components in one formulation such as two microbial biocontrol agents (such as bacteria and fungi), a chemical pesticide and biocontrol agent or a biocontrol agent and efficacy augmenter agents (such as nanoparticles). Owing to improvements in polymer sciences, new polymer gels will have direct effect on capsule morphology hope fully resulting in excellent multi-compartment capsules. With attention to this technique, more try must be put into adapting formerly present techniques to usage of biocontrol microorganisms. This may contain intelligent mixture of formerly techniques to dissolve several problems of biocontrol microorganisms such as shelf life. In future, this technology, considering all its advantages, including the gradual release of beneficial microorganisms, can create a new path in the formulation of antagonistic agents.

## Figures and Tables

**Table 1:** Different carrier materials and methods used in encapsulation of microbial cells.

Carrier	Method	Microorganism	References
Malt dextrin	Spray drying	*Beijerinckia* sp.	[56]
Whey and skim milk	Spray drying	*Lactobacillus acidophilus*	[57]
Whey protein	Spray drying	*Bifidobacterium breve*	[58]
Corn flour	Spray drying	*Bacillus thuringiensis*	[59]
Gum Arabic	Spray drying	*Trichoderma harzianum*	[60]
Chitosan-gellan gum	Spray drying	*Streptomyces fulvissimus* Uts22	[61]
Alginate-Gelatin	Emulsion	*Pseudomonasfluorescens* VUPF5	[42]
Alginate	Emulsion	*Bifidobacterium* BB-12	[62]
Alginate-bentonite	Extrusion	*Bacillus subtilis* Vru1	[53]
Maltodextrin-gum arabic	Spray Drying	*Bacillus cereus* C1L	[63]
Starch-pectin	Emulsion	*Bifidobacterium bifidum* F-35	[64]
Alginate, chitosan	Extrusion	*Lactobacillus casei* ATCC 393	[65]
Arabic gum	Spray drying	*Lactobacillus paracasei* NFBC 338	[66]
Psyllium-gum Arabic	Extrusion	*Enterococcus durans* IW3	[67]
Alginate-starch-bentonite	Extrusion	*R. planticola* Rs-2	[68]
Alginate	Extrusion	*Pseudomonas putida* CC-FR2-4 and *Bacillus subtilis* CC-pg104	[69]

**Table 2:** Some formulations of plant growth promoting rhizobacteria for using in plants.

Formulation	Additives or Treatment	Microorganism	Plant Species or Substrate	References
Alginate	Humic acid	-	-	[164]
Carboxymethyl cellulose/corn starch	Magnesium oxide	*Azospirillum brasilense, Burkholderia tropica*	Cowpea, Sugarcane	[165,166]
Alginate	Starch	*Raoultella terrigena, Azospirillum brasilene*		
Alginate-Bentonite- Starch	-	*Raoultella planticola*	-	[68]
Alginate- gelatin	-	*Bacillus subtilis*	-	[167]
Alginate- gelatin	Carbon Nanotubes and Silicon dioxide nanoparticle	*Pseudomonas fluorescens, Bacillus subtilis*	Potato	[42]
Alginate-Bentonite- Starch	titanium dioxide nano particle	*Bacillus subtilis*	Bean	[53]

## Data Availability

Not applicable.
